# A novel in vitro high-content imaging assay for the prediction of drug-induced lung toxicity

**DOI:** 10.1007/s00204-024-03800-8

**Published:** 2024-05-28

**Authors:** Paul A. Fitzpatrick, Julia Johansson, Gareth Maglennon, Ian Wallace, Ramon Hendrickx, Marianna Stamou, Kinga Balogh Sivars, Susann Busch, Linnea Johansson, Natalie Van Zuydam, Kelley Patten, Per M. Åberg, Anna Ollerstam, Jorrit J. Hornberg

**Affiliations:** 1 Safety Sciences, Clinical Pharmacology and Safety Sciences, R and D, AstraZeneca, Gothenburg, Sweden; 2 AstraZeneca Pathology, Clinical Pharmacology and Safety Sciences, R and D, AstraZeneca, Cambridge, UK; 3 Drug Metabolism and Pharmacokinetics, Research and Early Development, Respiratory and Immunology (R and I), R and D, AstraZeneca, Gothenburg, Sweden; 4 Data Sciences and Quantitative Biology, Discovery Sciences, R and D, AstraZeneca, Gothenburg, Sweden

**Keywords:** Occludin, High-content imaging, Drug-induced toxicity, Discovery safety, Inhalation toxicity

## Abstract

The development of inhaled drugs for respiratory diseases is frequently impacted by lung pathology in non-clinical safety studies. To enable design of novel candidate drugs with the right safety profile, predictive in vitro lung toxicity assays are required that can be applied during drug discovery for early hazard identification and mitigation. Here, we describe a novel high-content imaging-based screening assay that allows for quantification of the tight junction protein occludin in A549 cells, as a model for lung epithelial barrier integrity. We assessed a set of compounds with a known lung safety profile, defined by clinical safety or non-clinical in vivo toxicology data, and were able to correctly identify 9 of 10 compounds with a respiratory safety risk and 9 of 9 compounds without a respiratory safety risk (90% sensitivity, 100% specificity). The assay was sensitive at relevant compound concentrations to influence medicinal chemistry optimization programs and, with an accessible cell model in a 96-well plate format, short protocol and application of automated imaging analysis algorithms, this assay can be readily integrated in routine discovery safety screening to identify and mitigate respiratory toxicity early during drug discovery. Interestingly, when we applied physiologically-based pharmacokinetic (PBPK) modelling to predict epithelial lining fluid exposures of the respiratory tract after inhalation, we found a robust correlation between in vitro occludin assay data and lung pathology in vivo, suggesting the assay can inform translational risk assessment for inhaled small molecules.

**Supplementary Information:**

The online version contains supplementary material available at 10.1007/s00204-024-03800-8.

## Introduction

Inhaled drug delivery is an effective treatment paradigm for respiratory diseases, such as asthma and chronic obstructive pulmonary disease (COPD), due to direct access to the intended target organ and the potential to minimize systemic exposure and consequent side effects (Ruge et al. 2013). The development of innovative inhaled medicines is often impacted by toxicity in the respiratory tract in non-clinical species, leading to attrition or dose limitations in clinic, typically after the selection of novel drug candidates (David et al. 2014). As part of the industry shift from observational to predictive toxicology, a proactive discovery safety strategy aims to identify and mitigate these safety risks early during drug discovery, and thereby enable the design and selection of drug candidates with the right safety profile (Hornberg et al. 2014a; Hornberg and Mow 2014; Morgan et al. 2018). This relies heavily on in vitro methods that are predictive for in vivo toxicity, clinical safety and compatible with the design-make-test-analyze (DMTA) cycle during lead optimization campaigns (Hornberg et al. 2014b; Johansson et al. 2019). A plethora of high-content screening (HCS) methodologies have been developed and deployed to successfully identify for example cardio-, hepato-, neuro-, and nephrotoxicity (Li and Xia 2019; Persson and Hornberg 2016). However, the development of suitable in vitro assays to predict inhaled toxicity has been a challenge, which is due in part to the different routes of administration, inhaled versus systemic but also the complexity of the human lung. The lung is composed of approximately 50 different cell types at various stages of differentiation within the lung epithelia and involves a complex structure combining air conducting and gas exchanging regions (Berube et al. 2010; Hiemstra et al. 2018). The primary target within the lung for inhaled toxicants is the epithelial layer (Hiemstra et al. 2018). The tissues of the conducting airways are lined by a layer of pseudostratified columnar epithelium containing ciliated, goblet, club and basal cells. This epithelial layer has a protective function, but also moistens inhaled air (Vielle et al. 2019). The alveolar space is composed primarily of alveolar type I and II epithelial cells. Type I alveolar cells form a single layer which allows rapid gas exchange with the bloodstream while type II produces surfactants and gives rise to new alveolar cells (Knudsen and Ochs 2018). In order to prevent the passage of external detritus into the bloodstream, these epithelial cells form a tight cell-to-cell junction that acts as a barrier (Overgaard et al. 2012). The integrity of the epithelial barrier in the lung is often compromised in respiratory diseases, which can be studied in vitro using complex air–liquid interface (ALI) cultures of primary human epithelial cells (Aghapour et al. 2018; Gon and Hashimoto 2018; Hiemstra et al. 2018; Pell et al. 2021; Upadhyay and Palmberg 2018). We have previously shown that measurements of trans-epithelial electrical resistance (TEER) and cell viability in such a physiologically relevant trans-well model are highly predictive for respiratory toxicity of inhaled drugs (Balogh Sivars et al. 2018). The ALI model has, however, limitations for applicability in early drug discovery decision-making. Due to its complexity and consequent resource intensity, the ALI model does not have sufficient throughput to test large numbers of compounds to inform dose response and structure–activity relationships. In addition, we have previously shown that TEER measurements in ALI models appear sensitive only at a high concentration (400 µM, single dose level) which presents issues with precipitation for poorly soluble inhaled small molecules. Compounds are therefore typically scored categorically as toxic or non-toxic, whereas lead optimization campaigns rely on quantitative information, such as half-maximal inhibitory concentration (IC_50_) values in a relevant concentration range. The maintenance of these physiological barriers, often measured via TEER, including the air–blood, blood–brain and intestinal barriers, is supported primarily by junctional proteins (Hashimoto and Campbell 2020; Rouaud et al. 2020; Slifer and Blikslager 2020). We hypothesized that high-content imaging of tight junction proteins in a lung epithelial 2D culture would enable a scalable alternative to the ALI cultures, amenable for use during the drug discovery phase and for quantitative risk assessment.

Here we developed a novel high-content screening assay, based on the quantification of the tight junction protein occludin in A549 cells, as a surrogate marker for lung epithelial barrier integrity and consequent pulmonary toxicity. We validated the predictivity of the assay using 19 inhaled small molecules with known lung safety profiles, based on non-clinical and clinical data and demonstrated how the assay could be applied to quantitative risk assessment. This method provides a cheap, fast and highly predictive way to screen early discovery phase compounds for identification of undesirable safety profiles to guide chemistry design.

## Materials and methods

### Cell culture

A549 (ATCC), an alveolar epithelial cell line, and CALU-3 (ATCC), an epithelial cell line, were cultured in Dulbecco’s modified Eagle’s medium (DMEM) supplemented with 10% foetal bovine serum (FBS). 16HBE bronchial epithelial cells, kindly provided by Carl Staples (University of Southampton), were cultured in DMEM supplemented with 10% FBS and 1% non-essential amino acids). All culture media materials were purchased from Thermo Fisher Scientific. For imaging experiments, cells were seeded at 10,000 cells per well in 96-well CellCarrier imaging plates (Perkin Elmer) and cultured for 72 h to allow tight junction formation. All experiments were carried out between passages 3 and 15.

### Compound treatment

All tested compounds were synthesized internally and provided in 100% DMSO. Compounds were diluted in appropriate media to a 9-point concentration range from 100 µM down to 15 nM with a final concentration of 1% DMSO. Cigarette smoke extract (CSE) was produced by passing smoke from 5 University of Kentucky research cigarettes with filters removed through PBS with an air pump at a rate 0.07 L/min. CSE was diluted in appropriate media to a concentration range of 12% to 0.01%. TNF-α and TGF-β (Thermo Fisher Scientific) were diluted in appropriate media to a concentration range of 50 ng/ml to 0.02 ng/ml. All treatments were for 24 h and terminated by removing media and fixing with 4% paraformaldehyde (PFA). Vehicle (1% DMSO) was included for each plate and all treatments were carried out in duplicate on each plate. All experiments were independently repeated at least 3 times.

### Immunofluorescence imaging

Following treatment, cells were washed twice in PBS and fixed in 4% PFA for 20 min at room temperature and permeabilized using 0.5% Triton-X for 15 min at room temperature. Cells were then blocked using 5% goat serum in PBS at room temperature for 1 h. Primary antibodies were applied as per optimized conditions based on manufacturers recommended dilutions overnight at 4 °C. Cells were washed twice in PBS prior to application of secondary antibodies at a dilution of 1:5000 for 1 h at room temperature. Cells were washed twice with PBS and subsequently stained with a mixture of Hoechst and Cell Mask Deep Red at 1:1000 in PBS. Finally, cells were washed twice in PBS and either stored at 4 °C or imaged immediately. Plates were imaged using a MolecularDevices IXC. Each well was imaged at 40 × magnification with 16 images taken per well which provided 32 images per condition for each biological replicate. Cell viability was assessed through direct counts of nuclei.

### Image analysis

Analysis modules were written using the custom module editor of MetaXpress version 6.5.4.532. Nuclei were segmented and enumerated using the embedded count nuclei module. Cellular membrane outlines and areas of cellular junctions were identified using the Cell Mask and anti-occludin antibody staining, respectively. Briefly, due to the propensity of this dye to stain all cellular structures, the Cell Mask image was inverted to better highlight the plasma membrane staining. The resulting image was watershed using the previously identified nuclei. This watershed image was then again inverted to give a 1 pixel outline of all cell membranes. In order to fully capture the area of cell membrane responsible for tight junctions, this membrane area was expanded using the Grow Objects function to incorporate a space of 10 pixels on either side of the identified membrane. This thickened outline mask was then applied to the cell mask image and the area of staining was quantified. For each image, the area of membrane staining is reported as the total for all cells within the entire image. Tight junction staining was assessed in a similar manner; however, image inversion was not required due to the significantly more specific staining pattern. To quantify the amount of occludin staining at the membrane, the final step of the module overlaid the thickened membrane mask with the thickened junction mask. The occludin staining fluorescence area within this image overlay was quantified as a measure of tight junction presence and compared between experimental conditions to assess drug-induced effects on tight junctions. Within each image, all nuclei were counted and expressed as a mean value of all 16 images for each well. Loss of cell viability was determined as a drop in total cell nuclei counts. Area of membrane and area of occludin staining in µm^2^ per image were expressed as the mean of all 16 images normalized to the number of cells counted.

### Physiologically based pharmacokinetic modelling

A whole-body rat-specific PBPK model, which places emphasis on pulmonary drug disposition, was implemented in MATLAB R2017a (MathWorks Inc., Natick, MA, USA) (Boger et al. 2016; Boger and Friden 2019). The lung was divided into 24 airway generations and one extra-thoracic region according to the morphometry presented by (Lee and Wexler 2011). The structural model is illustrated in supplementary Fig. 1, where airway generation 1 refers to the trachea. Details regarding calculations of the regional surface areas as well as mucociliary clearance and volumes of the epithelial lining fluid (ELF), epithelium and sub-epithelium are provided in (Boger et al. 2016; Boger and Friden 2019). Each airway generation is divided into three compartments: (1) ELF, (2) epithelium and (3) sub-epithelium (supplementary Fig. 1). The airway generations either belong to the tracheobronchial- (generation 1–16) or the alveolar region (generation 17–24). Drug particle dissolution (if applicable) in the ELF was modelled by the Nernst–Brunner equation (Nernst 1904; Noyes and Whitney 1897). In addition, the model assumed the absence of any non-specific binding in the ELF compartment. Perfusion-rate limited distribution was assumed to apply for all tissues in the PBPK model. An additional tissue-binding compartment was introduced in each epithelium and sub-epithelium compartment with model optimized values for k_in_ and k_out_ representing the distribution in and out of a volume-less ‘deep’ compartment. The permeability in the alveolar region was set at tenfold, the value for the bronchiolar region. The tracheobronchial region is perfused by the bronchial blood flow and the alveolar region by the entire cardiac output. The local bronchial blood flow to a tracheobronchial generation was calculated according to (Boger et al. 2016; Boger and Friden 2019). The local blood flow to the alveolar generations was assumed to be constant in terms of flow per tissue volume. All ordinary differential equations (ODEs) used in the model are provided in (Boger et al. 2016; Boger and Friden 2019).

The PBPK model was first fitted to available plasma and lung concentrations after intratracheal dosing with 70 and 30% deposition in the tracheobronchial- and alveolar region, respectively (Boger and Friden 2019). The resultant optimized values for the main PBPK model parameters whilst setting constant the values for the systemic PK parameters of CL and V_ss_ can be found in supplementary Table 1. The optimized PBPK model was subsequently used to simulate the unbound compound concentrations in the epithelium lining fluid for all tracheobronchial generations (Gen. 1–16) after dry powder inhalation.

### Nonclinical toxicology study and histopathological assessment

Inhalation dosing of the small molecule AZ5 was achieved using a dry powder aerosol, generated using a Wright Dust Feed (WDF) mechanism. The exposure system comprised an aerosol conditioning pre-chamber and a snout-only inhalation exposure chamber, to which Han Wistar rats were attached via restraining tubes, air supply and extract lines. The duration of dosing was 30 min, once per day for a period of 4 weeks. The achieved dose levels were 0.361, 2.20 or 13.8 mg/kg/day (target dose levels of 0.3, 2 or 15 mg/kg/day, respectively), using aerosol concentrations of 16.2, 99.0 or 621 µg/L. The mass median aerodynamic diameters (MMAD) were within the respirable range (1 to 3 µm) at all dose levels. During the course of the study, blood samples were taken on days 1 and 28, immediately post-dose and up to 23.5 h post-dose to measure plasma exposures. Compound levels in plasma were used in the PBPK modelling for calculating ELF concentrations.

At study termination, all animals underwent a full necropsy examination and selected tissues were collected for histopathological examination. Tissues collected from the respiratory tract included nasal cavity, larynx, trachea, carina, extrapulmonary bronchus and lung. Tissues were collected into 10% neutral-buffered formalin, processed and embedded in paraffin and sectioned on a microtome according to standard methods. Sections were stained with haematoxylin and eosin and examined by light microscopy.

### Data analysis and statistics

IC_50_ values for each in vitro treatment were calculated using GraphPad prism version.8.4.2. Sensitivity, specificity and accuracy (overall percentage of correct predictions) of the assay were calculated according to Cooper statistics (Cooper et al. 1979). Statistical significance of validation compound concentrations was calculated as a Student’s *t* test in GraphPad prism version.8.4.2 from three biological replicates.

## Results

### Measurement of junctional staining and treatment-driven perturbations in epithelial cells

**Fig. 1:**
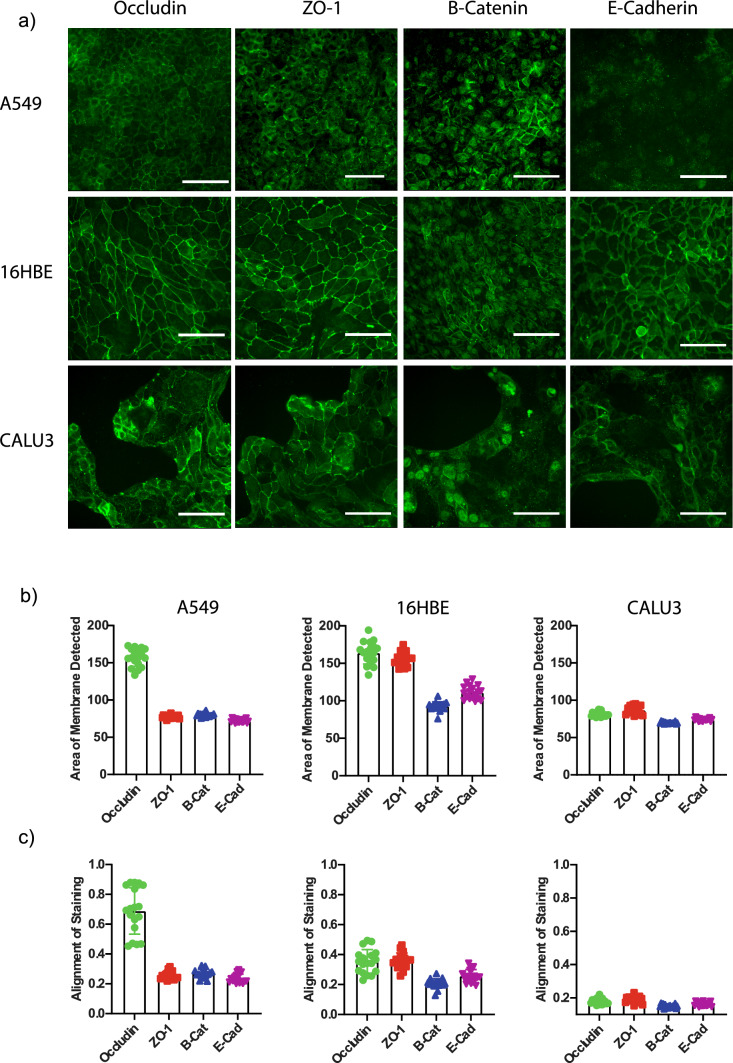
Characterization and measurement of junctional staining in multiple epithelial cell lines. **A** A549, 16HBE and CALU-3 stained for Occludin, ZO-1, β-catenin and E-cadherin. Scale bar represents 200 µm. **B** Area of membrane staining detected for each antibody was measured within each cell line. Results are expressed as the mean of the area (µm^2^) (*n* = 96, error bars represent SD). **C** Area of junctional staining was aligned with the area of membrane staining to ascertain the most robust cell line and stain combination. Results are expressed as the % alignment between both staining regions

To establish the screening platform, we aimed to select an appropriate combination of a tight junction marker and a cell line that would be compatible with high-throughput image analysis as well as respond to relevant stimuli. We characterized antibodies for four junctional proteins (occludin, ZO-1, β-catenin, E-cadherin) across three commonly used respiratory epithelial cell lines (A549, CALU-3, 16HBE). Of the cell lines explored, CALU-3 cells were shown to have the greatest degree of variability in their growth pattern with a tendency to grow in 3D rather than in a single monolayer. Three-dimensional cultures can influence staining and imaging analysis in a multi-well format, introducing variability. For CALU-3, this resulted in irreproducible, poor-quality images across all markers tested and therefore not of sufficient quality for further assay development. 16HBE cells displayed a robust growth pattern with well-defined junctional borders for occludin and ZO-1. However, these borders did not overlap consistently with cell edges as detected by the cell mask staining, which hampered attempts to quantify changes. A549 cells however, had a consistent growth pattern, achieving a single monolayer over similar incubation times. Whilst the ZO-1, β-catenin and E-cadherin staining for these cells was either not reproducible or lacked the expected staining pattern, occludin displayed the expected “cobblestone” staining pattern (Fig. 1a). This allowed for robust quantification of the junctional area stained per image (Fig. 1b) and the alignment of junctional markers to the cell membrane region (Fig. 1c).

**Fig. 2:**
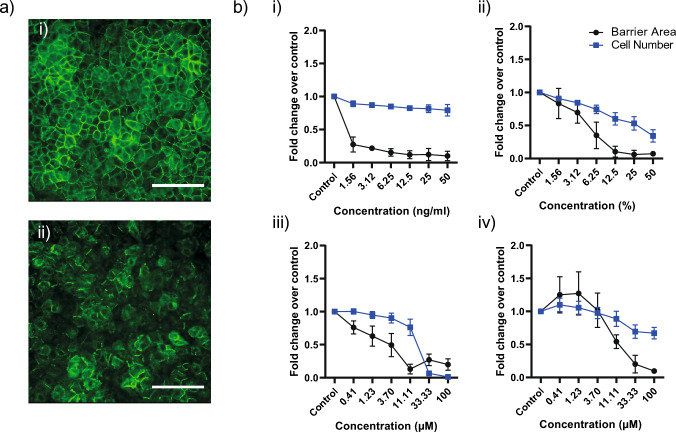
Perturbation of barrier phenotype with known inducers of lung epithelial barrier impairment accurately measured as a reduction in junctional occludin staining. **A** Imaging of occludin staining in cells treated with (i) DMSO and (ii) CSE show a significant breakdown in the staining pattern following treatment. **B** Treatment of A549 cells with a combination of (i) TNFα/TGFβ (ng/ml), (ii) CSE (%), (iii) CdCl2 (μM), and (iv) an internal small molecule with defined in vivo lung pathology reduced the area of junctional staining in A549 cells across concentrations independent of changes in cell number

Next, we determined whether occludin staining in A549 cells could detect perturbations of the cell-to-cell junctional barrier. Cells were treated with known modulators of lung epithelial barrier integrity: CSE, Cadmium chloride, TNF-α, TGF-β and a small molecule IKK2 inhibitor (Balogh Sivars et al. 2018; Schilpp et al. 2021). Image analysis detected a significant decrease in the area of junctional staining present at the cell membrane in treated wells compared to DMSO controls, in a concentration-dependent manner (Fig. 2). The staining was not only associated with cell loss, which was clearly demonstrated using CSE. This indicated the screening system could indeed detect induced changes to the cell barrier staining pattern.

### Prediction of lung irritancy for inhaled small molecules

In order to establish the assays predictive performance for drug-induced lung toxicity, a set of 19 inhaled small molecules were tested across a 9-point concentration range up to 100 µM, for their ability to induce changes to the occludin marker in the junctional area. The compound set included a mix of 10 compounds that had previously demonstrated irritancy in the respiratory tract (group 1), at relevant dose levels in pivotal toxicology studies in pre-clinical species or in the clinic, as well as 9 compounds that had a suitable lung safety profile to support clinical development or market registration (group 2) (Table 1).

**Table 1:** Compound Assay Validation Set

Label	Mechanism	Respiratory safety risk	IC_50 (_µM)	Comment
AZ1	Tyrosine Kinase inhibitor	Yes	11.0	Lung inflammation, hyperplasia and increased lung weight in rat and dog
AZ2	Tyrosine Kinase inhibitor	Yes	NA	Lung inflammation and hyperplasia in rat and dog
AZ3	Protein Kinase inhibitor	Yes	9.6	Inflammatory epithelial pathology in rat and dog
AZ4	Antimicrobial	Yes	16.8	Marketed product with increase cough and difficulty breathing
AZ5	Tyrosine Kinase inhibitor	Yes	27.9	Epithelial atrophy/degeneration progressing to erosion/ulceration in upper/central airways in rat
AZ6	Adrenergic receptor agonist	Yes	35.3	Subacute respiratory tract irritancy in rat
AZ7	Tyrosine Kinase inhibitor	Yes	46.7	Lung inflammation and hyperplasia in rat
AZ8	Phosphodiesterase antagonist	Yes	36.4	Bronchopneumonia / rhinitis in dog
Cadmium chloride	NA	Yes	3.7	Pneumonitis and fibrosis
AZ10	Phosphoinositol antagonist	Yes	2.1	Inflammatory cell infiltrates, alveolar macrophage aggregates with degenerative cytomorphology in rat
AZ11	Mast cell stabilizer	No	NA	Marketed product
AZ12	Muscarinic acetylcholine receptor antagonist	No	NA	Marketed product
AZ13	Acetylcholine receptor antagonist	No	NA	Marketed product
AZ14	Adrenergic receptor agonist	No	NA	Marketed product
AZ15	Muscarinic acetylcholine receptor antagonist	No	NA	Marketed product
AZ16	Adrenergic receptor agonist	No	NA	Marketed product
AZ17	Serine Proteinase antagonist	No	NA	Marketed product
AZ18	Muscarinic acetylcholine receptor antagonist	No	NA	Lung function changes in animals without pathology, reduced FEV1 in humans
AZ19	Antibiotic	No	NA	Marketed product

Compound table including a mix of compounds with demonstrated irritancy in the respiratory tract (group 1), and 9 compounds that had a suitable lung safety profile to support clinical development or market registration (group 2)

**Fig. 3:**
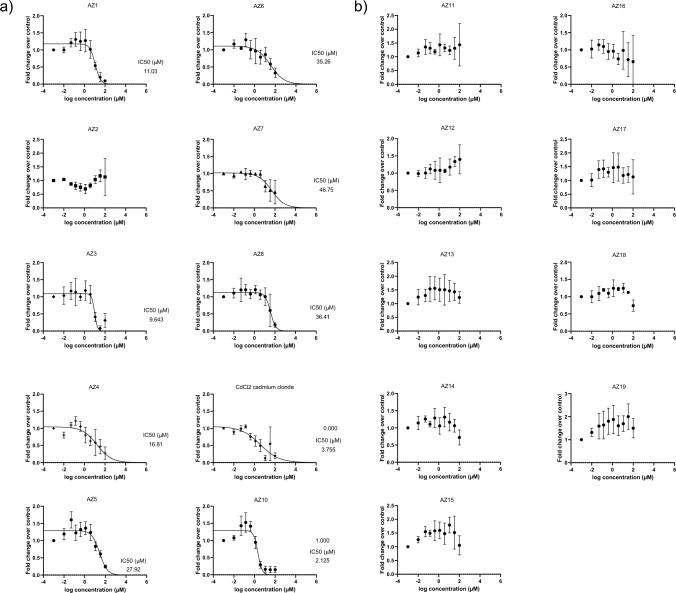
IC_50_ Curves generated from in-house compounds with a known (non)clinical lung safety profile. Results are expressed as fold change over control (*n* = 3 error bars represent SD) (**A**). IC_50_ curves for detected toxic compounds (**B**). Data from negative compounds with no curve generation possible

Occludin area staining was calculated for all compounds across a screening assay compatible concentration range. Values were normalized to total cell number and to DMSO controls. Linear regression analysis was used to generate individual IC_50_ values for compound ranking. For 9 of the 10 group 1 compounds, a clear effect on occludin staining could be detected and dose response curves could be established with IC_50_ values in the range 2–50 µM (Fig. 3a). The data also show the assay can rank compounds in terms of their response, which is highly advantageous in order to inform compound selection and chemical design. Indeed AZ1, AZ2 and AZ7 represent alternative chemistries with the same target with known differences in their in vivo safety profiles (data not shown) the severity of which aligns with the assay IC_50_. In contrast, some of the 9 group 2 compounds, namely AZ14 and AZ18 had only minor effects on Occludin levels at the highest concentration. Hence, IC_50_ curves could not be established for these compounds (Fig. 3b), clearly indicating the assay can distinguish between compounds with lung irritancy potential and those without.

**Fig. 4:**
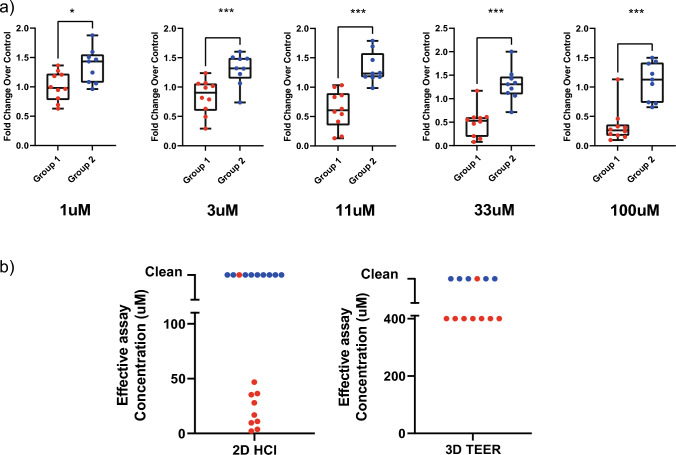
The occluding HCI assay could predict lung irritancy at lower concentrations compared to TEER measurements in ALI culture. **A** Significant differences between compounds with (group1) and without (group 2) a respiratory safety risk were seen across the entire assay concentration range. **B** In comparison with ALI-based TEER measurements, the imaging assay provides granularity between compounds with varying IC_50_ values in a relevant concentration range to support medicinal chemistry optimization programs

To explore the assay’s predictive ability across concentrations, Student’s t-test statistical analysis was performed on each concentration level. It clearly demonstrated that significant changes between respiratory irritants and non-irritants were detected at concentrations as low as 1 µM (Fig. 4a). When compared to previously published lung epithelial models (Balogh Sivars et al. 2018), the occludin imaging assay in A549 cells had similar predictivity using a comparable validation compound set with 90% sensitivity and 100% specificity. However, that sensitivity was achieved at a lower concentration range (Fig. 4b). Additionally, due to the 96-well plate format, the simple cell system and quick assay protocol, the assay has sufficient throughput to easily obtain full dose–response curves.

### Alignment of predicted ELF concentrations and assay IC_50_ with observed pathological findings

**Fig. 5:**
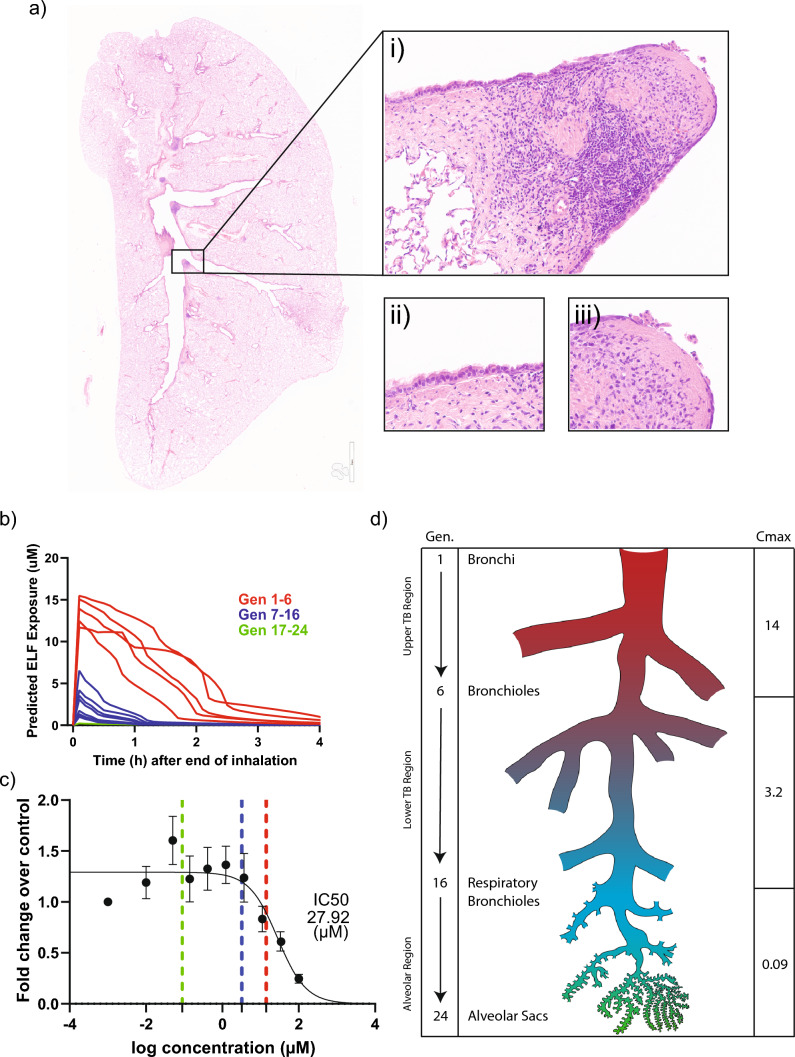
In vitro assay data align with predicted ELF C_max_ in regions with and without lung pathology from a rat dry powder inhalation study. **A** H&E (i) images display areas of progressive loss of cilia (ii) and epithelial erosion (iii) in the bronchial bifurcation regions and are representative of areas within the upper tracheobronchial region where lesions were commonly located. **B** PBPK modelling predicted compound concentration in the ELF across the generations of the lung over time. **C** C_max_ concentrations from generations 1 to 6 (red), 7 to 16 (blue), and 17 to 24 (green) are plotted onto the imaging assay IC_50_ curve for the same compound. Results are expressed as fold change over control. **D** Graphic shows the calculated C_max_ concentrations in each lung region

With the development of the assay and validation of its predictivity, we established a tool to identify the hazard of lung toxicity for inhaled small molecules and to support the generation of structure–activity relationships required to optimize chemical series to remove that hazard. Next, we sought to examine if the assay had applicability for quantitative risk assessment. As a proof of concept, we assessed how the safety profile of an inhaled small molecule candidate drug from our internal compound collection could be quantitatively predicted by the occludin imaging assay. In a dry powder inhalation toxicology study with AZ5 in rats, we observed pathological changes consistent with airway irritation, with areas of cilia loss, epithelial erosion and ulceration (Fig. 5a). These pathological changes to the rat lung were restricted to the upper tracheobronchial (TB) areas, where compound concentrations are typically higher than in the lower areas of the respiratory tract. At the highest dose, 19 out of 20 animals displayed either minimal (10) or mild (9) tracheal epithelial alteration (loss of cilia and flattening of the epithelial cells). Eight animals experienced minimal (4), mild (3) and moderate (1) epithelial alteration at the carina, and 6 animals had a minimal severity mixed inflammatory cell infiltrate. In the lung, epithelial alterations were also found in the bronchi of 15 animals classified as minimal (12) or mild (3) severity, whilst 5 animals experienced minimal (3) or mild (2) bronchial erosion and one had minimal severity bronchiolo-alveolar metaplasia/hyperplasia. Aggregates of alveolar macrophages, minimal (8) or mild (5) were found in 13 animals.

Plasma exposures were fed into a PBPK model, which incorporates various compound-specific properties, such as distribution, solubility, dissolution of the dry powder, and aspects of the lung tissue architecture, such as cell type permeability (Boger et al. 2016), to calculate the ELF concentrations of the compound in the various areas (generations) of the respiratory tract over time following inhalation (Fig. 5b). This yielded relevant maximum exposure averages for the upper TB, lower TB and alveolar regions of the respiratory tract (Fig. 5d). The occludin results from the in vitro model were correlated to in vivo findings by mapping the modelled ELF concentrations onto the dose response curve. Interestingly, we found that by mapping the modelled ELF concentrations onto the occludin dose–response curve, we could show that pathological changes in vivo were only observed in areas of the respiratory tract that were exposed to drug concentrations (14 µM) similar to that of the in vitro IC_50_ (27 µM) (Fig. 5c). Equally, no pathological changes were observed in areas of the respiratory tract that were exposed to concentrations (3.2 & 0.02 µM C_max_) that did not elicit an effect in the occludin assay. This suggests that a combination of PBPK modelling and this novel in vitro assay can inform translational and quantitative risk assessment for inhaled small molecules.

## Discussion

Pharmaceutical R&D productivity requires a proactive discovery safety strategy to identify and mitigate safety risks early to bring forward candidate drugs with the Right Safety profile (Hornberg and Mow 2014; Morgan et al. 2018). To inform decision-making during drug discovery, robust in vitro assays are required that are compatible with integration in the DMTA cycle namely being cost-effective, have sufficient throughput, and with readouts that are predictive for (non)clinical safety (Blass 2021; Hornberg et al. 2014b; Johansson et al. 2019; McKim 2010; Sanders et al. 2017; Szymanski et al. 2012). Currently, a range of organ-specific assays exist, which use a single cell type rather than attempting to fully recapitulate the complexity of the organ they represent, several of which are applicable to early drug screening, for example those aimed towards the cardiovascular system, liver and kidney (Gustafsson et al. 2014; Persson et al. 2013; Pognan et al. 2023; Pointon et al. 2017; Sjogren et al. 2018). Options to screen inhaled drugs for lung toxicity however have lagged behind. Some bronchial cell-based assays have shown some promise when grown in ALI conditions, such as our predictive TEER-based ALI assay for the detection of lung toxicants (Balogh Sivars et al. 2018) and an imaging-based assay correlating junctional protein staining with TEER measurements as a primary readout of lung barrier integrity (Pell et al. 2021). Recently published breathing lung-on-chip models have also shown significant promise as an in vitro tool to study inhalation toxicity (Sengupta et al. 2022). However, these assays come with significant limitations in terms of throughput, cost, and their ability to stratify compounds within a chemical series. Relevant comparative assay details are listed in Table 2. A549 cells have been widely used as a cell model to study toxicological responses, such as cytotoxicity, oxidative stress and inflammatory responses (Barosova et al. 2021). However, TEER values from these cells when elevated to ALI conditions are far lower than alternative alveolar cell lines such as the lentivirus immortalized hAELVi cells. Indeed hAELVi cells have also been shown to possess a more type I phenotype than the type II of the A549 cells and thus representing a larger proportion of the alveolar population (Kuehn et al. 2016). In an effort to develop an assay which can be rapidly deployed for screening within a DMTA cycle, we focused on measuring a single endpoint from a widely studied cell line which, whilst not recapitulating the full complexity of the lung alveolar space allowed for robust measurement of a physiologically relevant parameter such as junctional protein morphology. Here, we showed that quantification of occludin staining in A549 cells in a simple 2D format can detect lung toxicants/irritants with 90% sensitivity and 100% specificity after 24 h compound administration. This is likely driven by the central role played by junctional proteins in maintaining an effective epithelial barrier in the lung. Occludin is a classic tight junction protein which functions to link cells to adjacent cells and to the internal cytoskeletal structures (Kojima et al. 2013). It has been shown to have a clear link to barrier maintenance, ATP production and gene regulation (Castro et al. 2018), whilst studies with occludin knock-out mice have also shown chronic inflammation amongst other pathologies, indicating not only a wide role for occludin within cellular homeostasis but also a pivotal link to inflammation and pathological processes (Saitou et al. 2000; Sugita and Kabashima 2020).

**Table 2:** Assay comparison to ALI-based TEER measurements

Factor	ALI TEER assay	Occludin imaging assay
Physiological relevance/cell types	Mixed bronchial cell types with multiple physiological functions largely focused on lung clearance	Single alveolar cell type with limited physiological functions focused on air/blood barrier integrity
Assay set up/turnaround time	20 + day differentiation required followed by a 10 day assay time with daily sampling and media change – 30 + Days	3 day passive culture period followed by 1 day testing and fixation – Imaging 2 day protocol – 7 Days
Predictive capacity	Sensitivity 88% Specificity 100%	Sensitivity 90% Specificity 100%
Labor intensity	50 Hours/Plate	10 Hours/Plate
Dosing required	Multiple	Single
Readouts	TEER value, LDH Release & Histopathology	Area of occludin staining in µm2 at cell interfaces
Testing range	Sensitive only at 400uM	Dose response sensitive 1-100uM
Decision making in drug discovery	Binary Value enabling hazard identification	IC50 enabling influence on chemical design during lead optimization
Quantitative risk assessment	No	Yes when combined with PBPK modelling
Automation compatible	No	Yes
DMTA Applicable	Low	High

Comparative assay table listing major differential factors in experimental design and readouts between previously published ALI-based TEER assay (Balogh Sivars et al. 2018) and the newly introduced occludin imaging assay

To develop an imaging-based assay measuring localization of a tight junction protein in a simple 2D culture, growth conditions of the chosen cell line were found to be of utmost importance. Specifically, in the development of this assay, it was found that the morphological characteristics of the bronchial cell lines 16HBE and Calu3 were incompatible with the requirements of the imaging algorithm. In order for robust measurement of the occludin staining pattern, it was necessary to use a whole cell stain to allow for determination of cell edges independent of the junctional marker to narrow the region of measurement to those edges. In the case of Calu3, these cells displayed a highly overlapping growth pattern, tending to form multilayered colonies rather than a uniform monolayer of cells. 16HBE, on the other hand, grew in a highly consistent monolayer fashion but displayed an undulating pattern of cell-to-cell contact rather than a liner line. These inherent conditions posed a challenge when it came to determining the degree of overlap of the junctional marker with the defined cell edge. Alternative seeding densities and culture time points were explored (data not shown). However, A549 cells were consistently found to provide the most robust basis to enable screening assay development.

In order to apply in vitro toxicology assays to quantitative risk assessment, it is essential to incorporate drug exposure information and establish how in vitro concentrations relate to clinical exposure (Bell et al. 2018). While there are challenges to scale compound concentrations and elicited effects in in vitro assays to plasma drug exposure and adverse events in the clinic, it has been possible to use in vitro assay data for quantitative risk assessment of some organ toxicities (Albrecht et al. 2019; Archer et al. 2018; Hengstler et al. 2020; Kappenberg et al. 2020; O'Brien et al. 2006; Persson et al. 2013; Sjogren et al. 2018). Quantitative risk assessment has been more challenging for inhaled drugs because, unlike for orally administered drugs, the systemic plasma exposure at a given inhaled dose does not accurately reflect the relevant exposure in the lung (Frohlich 2019; Kassinos et al. 2021). To address this gap, we applied an existing PBPK modelling approach, which mathematically describes respiratory tract physiology and the fate of deposited drug dose, and accurately predicts PK, target site exposure, and interestingly, spatial heterogeneity in target site concentrations within the lung (Boger et al. 2016; Boger and Friden 2019). This approach allowed us to directly correlate actual compound concentrations from the in vitro assay with calculated compound exposure in the ELF in a toxicity study and demonstrate that the pathological changes consistent with airway irritation were restricted to areas of the respiratory tract that reached concentrations that aligned with the IC_50_ of the imaging assay. While recent studies have shown the applicability of modelling to in vitro to in vivo extrapolation (IVIVE) for toxic vapours from agrichemicals (Moreau et al. 2022) and environmental contaminants such as endocrine disrupters (Xie et al. 2023), to our knowledge, this is the first-time in vitro toxicity data have been quantitatively linked to exposure levels in the lung for compound risk assessment in drug development.

The propensity of a compound to cause to toxicity may depend on primary target pharmacology, off-target secondary pharmacology and chemical reactivity, which originate from its chemical structure and physicochemical properties, and can manifest in many different downstream effects in the cell, including direct damage to organelles or DNA, membrane permeability, impaired cellular signalling or metabolism. In general, assessing cell health parameters has proven an effective trade-off between, on the one hand, ensuring high sensitivity (compared to more generic cell survival or cytotoxicity assessment) and, on the other hand, providing robust, accessible screening options with throughput (compared to more specific but therefore less-sensitive molecular endpoints). Here, the validation compound set encompassed diverse physicochemical properties, therapeutic targets, off-target pharmacology, and a range of pathologies across multiple pre-clinical tox species, including epithelial erosion, atrophy, oedema, hyperplasia and inflammation. In summary, we present a novel imaging-based assay that can be applied to identify and mitigate the risk for lung toxicity of inhaled compounds during early drug discovery, and for quantitative risk assessment in drug development. This will enable acceleration of the identification of inhaled drug candidates with the right safety profile without the need for animal studies.

### Supplementary Information

Below is the link to the electronic supplementary material.

Supplementary file1 (DOCX 143 KB)
