# Supplementary material for: A novel in vitro high-content imaging assay for the prediction of drug-induced lung toxicity
Source: Arch Toxicol. 2024 May 28;98(9):2985–98. doi: 10.1007/s00204-024-03800-8 (PMC11324770; doi:10.1007/s00204-024-03800-8)
Supplement: Supplementary file 1 — Supplementary file1 (DOCX 143 KB) [file 204_2024_3800_MOESM1_ESM.docx]

**Supplemental File 1**

**Title : A novel *in vitro* high content imaging assay for the prediction of drug-induced lung toxicity.**

**Journal :** Archives of Toxicology

**Authors & Affiliations:** Paul A. Fitzpatrick^1^, Julia Johansson^1^, Gareth Maglennon^2^, Ian Wallace^1^, Ramon Hendrickx^3^, Marianna Stamou^1^, Kinga Balogh Sivars^1^, Susann Busch^1^, Linnea Johansson^1^, Natalie Van Zuydam^4^, Kelley Patten^1^, Per M. Åberg^1^, Anna Ollerstam^1^, Jorrit J. Hornberg^1^.

^1^Safety Sciences, Clinical Pharmacology & Safety Sciences, R&D, AstraZeneca, Gothenburg, Sweden

^2^AstraZeneca Pathology, Clinical Pharmacology & Safety Sciences, R&D, AstraZeneca, Cambridge, UK

^3^Drug Metabolism & Pharmocokinetics, Research and Early Development, Respiratory and Immunology (R&I), R&D, AstraZeneca, Gothenburg, Sweden

^4^Data Sciences and Quantitative Biology, Discovery Sciences, R&D, AstraZeneca, Gothenburg, Sweden

Keywords: Occludin, High-content Imaging, Drug induced toxicity, Discovery safety, Inhalation toxicity

**Corresponding Author :** Paul Fitzpatrick **Email:** paul.fitzpatrick2@astrazeneca.com

**Supplementary Methods**

**Optimised PBPK values**

Supplementary Table 1

| Compound | Species | Delivered dose  dry powder inhalation (mg/kg) | MMAD (GSD)^¤^ (µm) | Solubility (µM) | Lung P_eff_  (1e-4 cm/s) | V_u lung_  (L/kg) | Dynamic lung tissue binding k_in_ / k_out_ (h-1) |
| --- | --- | --- | --- | --- | --- | --- | --- |
| AZ5 | Rat | 0.36 | 1.9 (1.9) | 18 | 5.0 | 1047 | 2.3 / 0.3 |
|  |  | 2.2 |  |  |  |  |  |
|  |  | 13.8 |  |  |  |  |  |

¤ MMAD: mass median aerodynamic diameter, GSD: geometric standard deviation

Rat PBPK model sized to dog, at 15 kg

**Tox study/pathology**

The *in vivo* toxicology study in the rat was conducted according to an authorized study protocol and local standard operating procedures in strict compliance with national legal regulations on animal welfare law and accepted animal welfare standards, with the numbers of animals and procedures used considered to be the minimum necessary to achieve the aims of the study.

Animals were housed in designated animal holding facilities, with appropriate control of temperature, humidity and light cycles; there were no significant deviations from the target ranges. Animals were group housed with environmental enrichment and were supplied with certified diet and water from the public supply.

**Supplementary Figure**


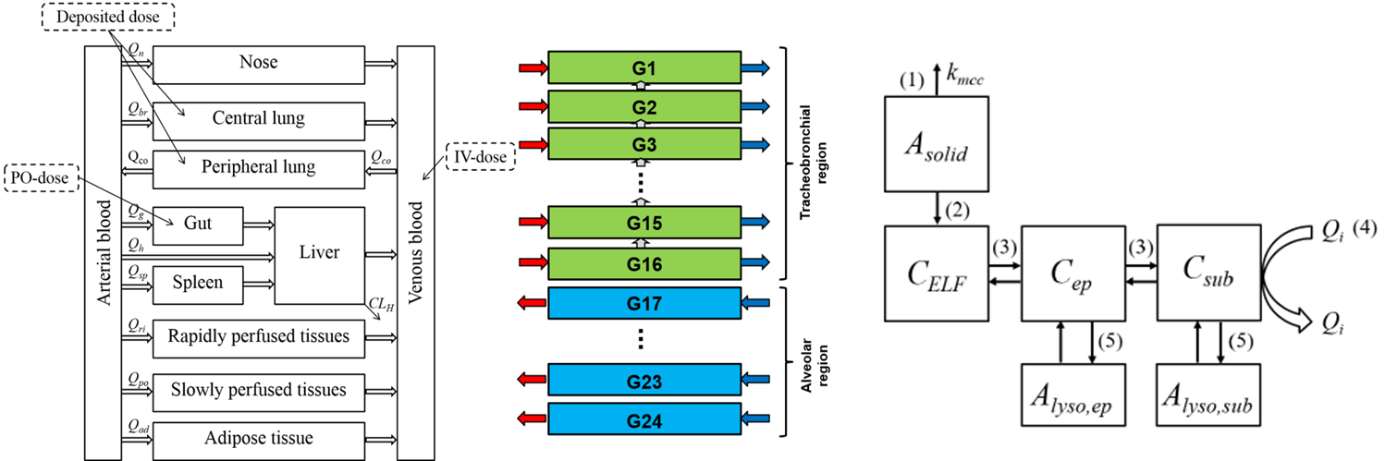


**S.Fig1.** Structure of the whole-body physiologically based pharmacokinetic model

Left panel: Structure of the whole-body physiologically based pharmacokinetic model. Middle panel: The lung was divided into 24 airway generations (Gen), which belong to either the tracheobronchial- (Gen1-Gen16, green) or the alveolar region (Gen17-Gen24, blue). Right panel: each airway generation was further divided into 3 main compartments: A) the epithelial lining fluid, B) the epithelium, and C) the sub-epithelium. The model accounted for the following processes in each generation after dosing: 1) Mucociliary clearance of solid in ELF in Gen1-Gen16, 2) Particle dissolution of solid in ELF, 3) Passive diffusion between ELF, epithelium and sub-epithelium, 4) Blood perfusion in sub-epithelium and 5) Deep tissue binding into non-voluminous compartment in both epithelium and sub-epithelium.
